# Human Primary Breast Cancer Stem Cells Are Characterized by Epithelial-Mesenchymal Plasticity

**DOI:** 10.3390/ijms22041808

**Published:** 2021-02-11

**Authors:** Juliane Strietz, Stella S. Stepputtis, Marie Follo, Peter Bronsert, Elmar Stickeler, Jochen Maurer

**Affiliations:** 1 Department of Immunology, University of Freiburg, 79104 Freiburg, Germany; juliane.strietz@biologie.uni-freiburg.de; 2 German Cancer Consortium (DKTK), DKFZ, 69120 Heidelberg, Germany; stella.stepputtis@gmail.com; 3 Department of Medicine I, Medical Center-University of Freiburg, University of Freiburg, 79106 Freiburg, Germany; marie.follo@uniklinik-freiburg.de; 4 Institute for Surgical Pathology, Medical Center-University of Freiburg, 79106 Freiburg, Germany; peter.bronsert@uniklinik-freiburg.de; 5 Department of Obstetrics and Gynecology, University Hospital Aachen (UKA), 52074 Aachen, Germany; estickeler@ukaachen.de

**Keywords:** triple-negative breast cancer, cancer stem cells, epithelial-mesenchymal transition, epithelial-mesenchymal plasticity

## Abstract

Triple-negative breast cancer (TNBC) is one of the most aggressive subtypes of breast cancer, with only limited treatment options available. Recently, cancer stem cells (CSCs) have emerged as the potential drivers of tumor progression due to their ability to both self-renew and give rise to differentiated progeny. The CSC state has been linked to the process of epithelial-mesenchymal transition (EMT) and to the highly flexible state of epithelial-mesenchymal plasticity (EMP). We aimed to establish primary breast cancer stem cell (BCSC) cultures isolated from TNBC specimens. These cells grow as tumor spheres under anchorage-independent culture conditions in vitro and reliably form tumors in mice when transplanted in limiting dilutions in vivo. The BCSC xenograft tumors phenocopy the original patient tumor in architecture and gene expression. Analysis of an EMT-related marker profile revealed the concomitant expression of epithelial and mesenchymal markers suggesting an EMP state for BCSCs of TNBC. Furthermore, BCSCs were susceptible to stimulation with the EMT inducer TGF-β1, resulting in upregulation of mesenchymal genes and enhanced migratory abilities. Overall, primary BCSC cultures are a promising model close to the patient that can be used both in vitro and in vivo to address questions of BCSC biology and evaluate new treatment options for TNBC.

## 1. Introduction

Breast cancer is the most frequently diagnosed cancer in women, and one of the leading causes of cancer death [1]. It is very heterogeneous in its histology, therapeutic response, metastatic behavior, and patient outcome. Among the various intrinsic subtypes [2], triple-negative breast cancer (TNBC) is the most aggressive one. It represents approximately 10–17% of all breast cancer cases [3]. TNBC is characterized by the lack of estrogen receptor (ER) and progesterone receptor (PR) expression, as well as by the absence of human epidermal growth factor receptor 2 (HER2) overexpression. Presenting no molecular weak points, tumors of this subtype are generally difficult to treat and thus have a poor prognosis [4,5].

For breast cancer [6] and various other cancers [7,8,9,10], it is hypothesized that tumor progression is driven by so-called tumor-initiating cells or cancer stem cells (CSCs). This small subset of cancer cells within the tumor has the ability to self-renew and thus expand the CSC population. It can further give rise to non-tumorigenic cells, and in this way shape the tumor with its heterogeneous cell lineages and architecture [11]. Some studies even suggest that this capacity to both self-renew and produce differentiated progeny may be comparable to the abilities of embryonic stem cells [12].

Experimental assays to confirm CSC presence and potential include transplantation into immunocompromised mice and in vitro surrogate assays such as anchorage-independent sphere assays [11].

So far, no universal cell surface marker combination has been identified that allows the clear distinction between tumorigenic BCSCs and non-tumorigenic breast cancer cells in all breast cancers, or even in a given subtype of breast cancer [13,14,15]. Nevertheless, different markers were proposed for BCSCs including CD44^+^/CD24^−/low^ [6], aldehyde dehydrogenase 1 (ALDH1) [16], CD49f [17,18], and EpCAM [6].

Moreover, various studies have suggested that the BCSC state can be linked to the epithelial-mesenchymal transition (EMT) state of the cell [16,19,20,21,22]. EMT is defined as a multifaceted and often reversible change in cellular phenotypes during which epithelial cells lose their apical-basal polarity, modulate their cytoskeleton and exhibit reduced cell-cell adhesive properties [23]. It was further described that cells can exist in intermediate states that are characterized by concomitant epithelial and mesenchymal characteristics [24]. This state has recently been referred to as epithelial-mesenchymal plasticity (EMP) [23], and there is the notion that BCSCs in this particular state display the greatest tumor-initiating capacity [16,25].

Conventional cell lines are the models that are most frequently used in breast cancer research. There are approximately 100 different cell lines available, although only a fraction of those are commonly used in published studies [26]. Furthermore, conventional breast cancer cell lines are reported to be relatively inefficient in xenograft tumor initiation [27,28], potentially due to low numbers of cancer stem cells in the cultures [29,30]. Since very few breast cancer models are enriched for BCSCs, there is a need for new reliable cell culture models to investigate this rare cell population in particular.

Here, we describe two new BCSC lines out of a panel of primary BCSC lines isolated from triple-negative breast cancer patient tumors. These cell lines are enriched for BCSCs due to the specific cultivation method used. They are highly tumorigenic and form tumors that recapitulate the architecture of the original patient tumor. Therefore, they present a promising tool to investigate BCSC characteristics and evaluate novel cancer therapies both in vitro and in vivo.

## 2. Results

### 2.1. BCSCs Can Be Isolated from Patient Tumor Samples

Over the last few years, we established a panel of primary breast cancer stem cell lines that we isolated from TNBC patient tumor tissues. These cell lines were already shown to be suitable tools to study TNBC in general, and cancer stem cells in particular [31,32].

**Figure 1:**
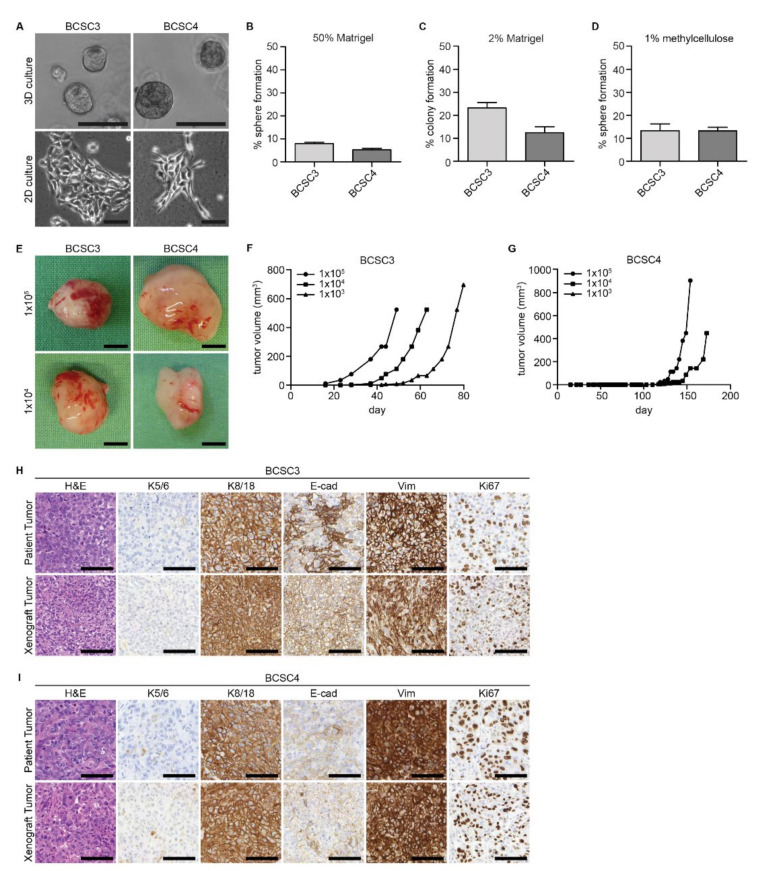
Characterization of BCSC3 and BCSC4 in vitro and in vivo. (**A**) Phase contrast images of BCSC3 and BCSC4 growing in MSC medium containing 50% Matrigel (3D culture) or 2% Matrigel (2D culture). Scale bars represent 100 μm. (**B**–**D**) Quantification of colony formation capacities of BCSC3 and BCSC4 single cells seeded in 3D in Matrigel (**B**), in 2D (**C**), or under anchorage-independent culture conditions (**D**). Spheres or colonies were counted. Values are sphere or colony formation relative to seeded cell number in percent (*n* = 3). Data represents means + SEM. (**E**) Images of representative BCSC3 and BCSC4 xenograft tumors generated from indicated cell numbers. Scale bars represent 5 mm. (**F**,**G**) Representative growth curves of BCSC xenograft tumors. BCSC3 and BCSC4 were transplanted orthotopically into NOD/SCID mice in limiting dilutions as indicated and the tumor size was measured over time (*n* ≥ 8 tumors per dilution). (**H**,**I**) Immunohistochemical analysis of BCSC3 and BCSC4 patient and xenograft tumors. Depicted are representative images of hematoxylin and eosin (H&E) staining as well as expression of keratin 5/6 (K5/6), keratin 8/18 (K8/18), E-cadherin (E-cad), vimentin (Vim), and Ki67. Scale bars represent 100 μm.

Similar to our previously published primary BCSC lines [31,32], BCSC3 and BCSC4 were cultivated in a 3D cell culture environment that is reported to be more physiological and selective for malignant cells. After this initial culture in 3D, the cells were cultivated in a 2D cell culture environment for more effective cell expansion (Figure 1A).

In a 3D cell culture environment using 50% Matrigel, BCSC3 and BCSC4 formed spheres anchorage-dependently with a respective sphere forming capacity of 7.9% and 5.2% (Figure 1B). In the 2D cell culture environment using 2% Matrigel, BCSC3 and BCSC4 formed adherent colonies with a colony formation capacity of 23.3% and 12.4%, respectively (Figure 1C). To further address the cancer stem cell potential in vitro, BCSC3 and BCSC4 were seeded in an anchorage-independent 3D sphere assay. There, the sphere formation capacities of BCSC3 and BCSC4 were at comparable levels at 13.3% and 13.2%, respectively (Figure 1D).

We further performed orthotopic transplantation of both primary BCSC lines in mice, which is the gold standard assay to confirm cancer stem cell abilities in vivo [11]. BCSC3 and BCSC4 both formed tumors when transplanted in limiting dilutions (Figure 1E). The tumor formation capacity differed slightly between both cell lines. Where BCSC3 reliably formed tumors from as few as 1 × 10^3^ transplanted cells (more than 90% tumor take rate), the tumor take rate of BCSC4 decreased with lower cell numbers. When 1 × 10^5^ cells were transplanted, the tumor take rate was 87.5% and decreased to 62.5% for 1 × 10^4^ transplanted cells. When as few as 1 × 10^3^ cells were transplanted, BCSC4 tumor formation was no longer detectable. Using ELDA analysis [33], we determined the BCSC content in our cultures to be 1 out of 434 cells for BCSC3 with a confidence interval of 1/974–1/194, and 1 out of 28,166 cells for BCSC4 with a confidence interval of 1/64357–1/12328.

Furthermore, the tumor onset varied between both cell lines. Whereas BCSC3 started to form tumors after approximately 18 days, BCSC4 exhibited a longer latency period and tumor formation was observed only after more than 110 days (Figure 1F,G, respectively). This in vivo growth behavior mirrors the in vitro growth, and the observed differences hint at the heterogeneity of the original patient tumors.

To address the question of whether BCSC-derived xenograft tumors can recapitulate the morphology and gene expression of the original patient tumor, isolated BCSC xenograft tumors were fixed, sectioned, and subjected to immunohistochemical (IHC) analysis (Figure 1H,I). BCSC3 and BCSC4 xenograft tumors morphologically resembled the patient tissue as determined by hematoxylin and eosin (H&E) staining. They also showed a similar proliferation index as visualized by Ki67 IHC. They were further analyzed for a marker panel including myoepithelial K5/K6, luminal epithelial K8/K18, epithelial E-cadherin, and mesenchymal vimentin. Similar to the corresponding patients, BCSC3 and BCSC4 tumors did not express myoepithelial K5/K6 but luminal epithelial K8/K18. Vimentin was strongly expressed for both, which was comparable to the corresponding patients. E-cadherin expression of BCSC3 xenografts varied slightly from the patient and for BCSC4, both patient and xenograft tumor only showed low expression of E-cadherin.

BCSC3 and BCSC4 xenograft tumors were further classified as triple-negative and thus mimicked the receptor status of the corresponding patient tumors (Figure S1).

We can therefore conclude that BCSC3 and BCSC4 are enriched with BCSCs and have the ability to recapitulate the original patient tumor histology from few transplanted cells.

### 2.2. BCSCs Exhibit Cellular Heterogeneity In Vitro

In breast cancer, heterogeneity is not only found between different subtypes, but also between tumors of the same subtype. This is reflected in the morphology of the different triple-negative BCSC lines that we describe here (Figure 1A) and in our previous publications [31,32].

Additionally, the cell lines provide insight into intratumoral heterogeneity in vitro since within the same cell culture, different types of colony morphologies were observed when single cells were seeded in a 2D culture environment (Figure S2). This observation highlights a retained cellular heterogeneity of the isolated primary cells within the cell line and further supports our technique of BCSC isolation and cell culture.

**Figure 2:**
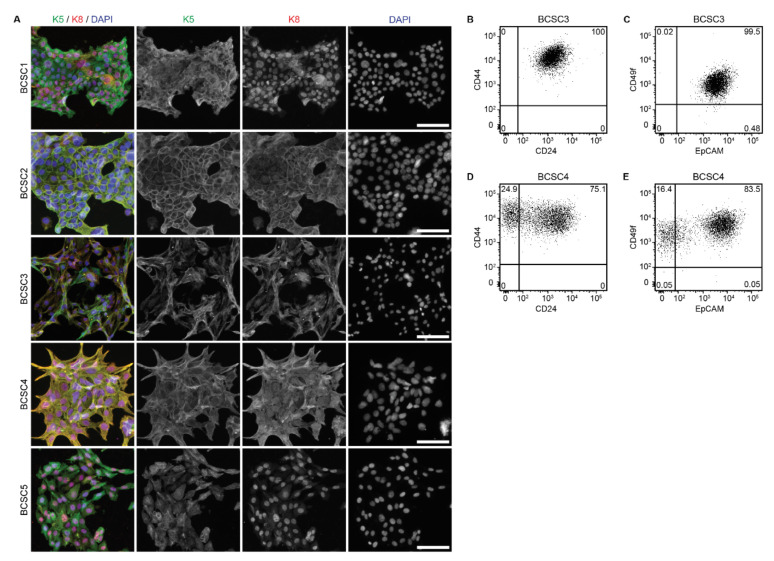
Breast cancer stem cells (BCSCs) exhibit cellular heterogeneity in vitro. (**A**) Immunofluorescence analysis of myoepithelial keratin 5 and luminal epithelial keratin 8 expression in BCSCs. Depicted are representative images. Nuclei were counterstained with DAPI. Merge and single channels of extended depth of focus z-stack images are shown. Scale bars represent 100 μm. (**B**–**E**) Flow cytometry analysis of BCSC surface marker expression in BCSC3 and BCSC4. Shown are representative expression patterns of CD24 and CD44 (**B**,**D**) as well as EpCAM and CD49f (**C**,**E**) (*n* = 3). Numbers represent the percentage of cells in the respective quadrant.

For a more in-depth characterization, the complete panel of five BCSC lines was examined regarding their expression of keratins, which are epithelial markers for cell lineage and differentiation status. Immunocytochemical (ICC) analysis showed that luminal epithelial K8 was detectable as intermediate filaments throughout the cytoplasm for all BCSCs, whereas myoepithelial K5 expression was present, but more heterogeneous between the different BCSC lines (Figure 2A). Additional analysis of myoepithelial K14 and luminal epithelial K18 confirmed these observations (Figure S3). This co-expression of myoepithelial and luminal epithelial keratins in all five BCSC lines suggests a bipotent precursor phenotype for BCSCs of TNBC with the capacity to generate both luminal and myoepithelial derivatives.

Since the cells were classified as BCSCs, we also analyzed the expression of proposed BCSC markers such as CD44, CD24, EpCAM, and CD49f using flow cytometry. For BCSC3, homogeneous marker expression was observed. 100% of cells were CD44^+^/CD24^+^ (Figure 2B), and 99.5% were EpCAM^+^/CD49f^+^ (Figure 2C). In contrast, marker expression of BCSC4 was more heterogeneous, as 24.9% of cells were CD44^+^/CD24^−^ and 75.1% were CD44^+^/CD24^+^ (Figure 2D). Furthermore, 16.4% of cells were EpCAM^+^/CD49f^−^ and 83.5% were EpCAM^+^/CD49f^+^ (Figure 2E). Similarly heterogenous results could be observed with the previously published BCSC lines [31,32].

Interestingly, although BCSC4 showed a higher percentage of CD44^+^/CD24^−^ cells compared to BCSC3, it showed lower colony formation capacity in vitro (Figure 1C) and slower tumor growth in vivo (Figure 1G). This observation cautions the sole use of surface marker expression for BCSC identification.

Since other studies suggest that BCSCs share functional similarities with embryonic stem cells [12,34,35,36], the expression of the stem cell-related transcription factors Sox2, Oct4, and Nanog was analyzed utilizing qRT-PCR. To illustrate the variability of these findings, all five BCSC lines are included here instead of only BCSC3 and BCSC4. We compared the expression of the three genes to mRNA from embryonic stem cells, as a strong positive control, and to mRNA of dermal fibroblasts, as a stem-cell marker negative control. Sox2 was expressed distinctly only in BCSC5 and lowly in BCSC2, whereas in BCSC1, BCSC3, and BCSC4, it was not expressed (Figure S4A). Oct4 was expressed moderately in BCSC1 and BCSC5 and hardly expressed in BCSC2, BCSC3, and BCSC4 (Figure S4B). Nanog was not expressed in any of the BCSC lines (Figure S4C). Overall, no or only very low expression of the embryonic stem cell markers Sox2, Oct4, and Nanog was detectable in primary BCSCs when compared to the expression in embryonic stem cells, and their expression did not correlate with the observed colony forming capacity or tumorigenicity. Nevertheless, as also shown by others [34,35,36], the stem cell markers were upregulated compared to non-cancer cells.

### 2.3. BCSCs Show an Intermediate EMT Phenotype

Over the last years, it was hypothesized that BCSCs exist in different EMT states [19,20] and that BCSCs expressing both mesenchymal and epithelial markers display the greatest tumor-initiating capacity [16,37]. To determine and compare the EMT state of the five BCSC lines, ICC, qRT-PCR, and Western blot analysis were used.

**Figure 3:**
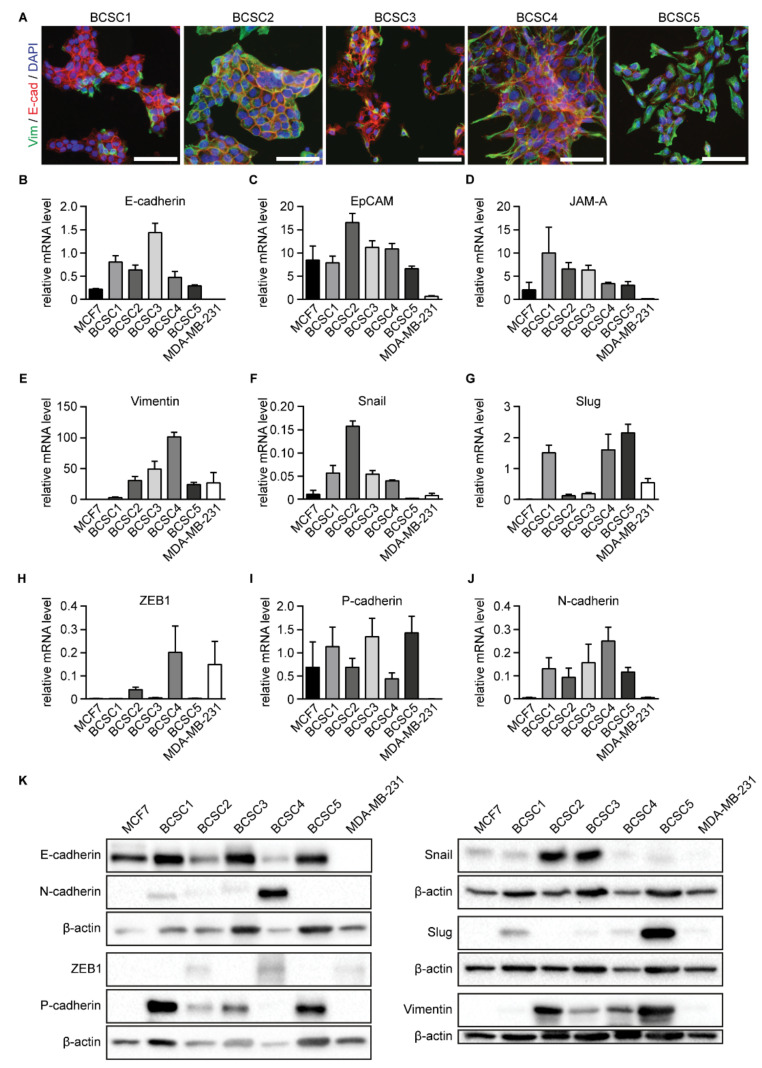
BCSCs co-express epithelial and mesenchymal markers. (**A**) Immunofluorescence analysis of BCSCs using antibodies against epithelial E-cadherin (red) and mesenchymal vimentin (green). Cell nuclei were counterstained with DAPI. Scale bars represent 100 μm. (**B**–**J**) Analysis of EMT-related gene expression in BCSCs at the mRNA level using qRT-PCR. MCF7 and MDA-MB-231 are depicted as points of reference. Shown are mRNA levels of E-cadherin (**B**), EpCAM (**C**), JAM-A (**D**), Vimentin (**E**), Snail (**F**), Slug (**G**), ZEB1 (**H**), P-cadherin (**I**), and N-cadherin (**J**) relative to HPRT1 (*n* ≥ 3). Data represents means + SEM. (**K**) Western blot analysis of epithelial and mesenchymal marker expression in BCSCs on the protein level. MCF7 and MDA-MB-231 are depicted as points of reference. β-actin served as loading control and is depicted for each individual Western blot membrane. Shown are representative blots (*n* = 3).

First, utilizing ICC, we visualized the expression of the mesenchymal marker vimentin and the epithelial marker E-cadherin in BCSCs cultured in a 2D culture environment. Interestingly, all BCSC lines co-expressed vimentin and E-cadherin in varying amounts. Whereas vimentin was expressed weakest in BCSC1, E-cadherin expression was weakest in BCSC5. All other BCSC lines showed substantial expression of both markers (Figure 3A and Figure S5).

Second, all BCSCs were screened for a broader mesenchymal and epithelial marker panel using qRT-PCR (Figure 3B–J) and Western blot analysis (Figure 3K). The epithelial breast cancer cell line MCF7 and the mesenchymal breast cancer cell line MDA-MB-231 were used as points of reference. The expression of specific markers varied between the different cell lines, giving them a distinct expression profile and again highlighting their heterogeneity. Nevertheless, all BCSCs co-expressed mesenchymal and epithelial markers simultaneously, suggesting an EMP state for triple-negative BCSCs. Similar expression of the EMT-related genes could be observed at the mRNA level when the cells were cultured in a 3D cell culture environment using 50% Matrigel (Figure S12).

Expression of EMT-related proteins such as Snail and Slug has been correlated with enhanced cell migration [38] whereas cells that express E-cadherin are often reported as non-motile [39]. To analyze how the EMP state is reflected in the migratory abilities of the BCSCs, scratch wound assays were performed. Results were documented for 48 h as relative wound density (RWD) in percentages for every point in time (Figure S6A–J). The observed migratory potential of the different cell lines varied considerably. BCSC1 and BCSC4 showed hardly any migratory ability, followed by BCSC5 and BCSC2, which showed only a slightly higher migratory potential. The highest migratory potential was observed for BCSC3. However, all BCSCs failed to completely close the scratch wound within 48 h.

### 2.4. Primary BCSCs Exhibit Cellular Plasticity

It was suggested that BCSCs can transition between a more mesenchymal-like (EMT) and a more epithelial-like (MET) state, and that this flexibility facilitates their capacity for migration and invasion [21,22]. Therefore, we investigated the cellular plasticity of the presented BCSCs using exposure to TGF-β1 to induce changes in the EMT state. Gene expression analysis using qRT-PCR showed that BCSCs express various genes related to the TGF-β signaling pathway and therefore can potentially be affected by soluble TGF-β1 in the surrounding microenvironment (Figure S7).

**Figure 4:**
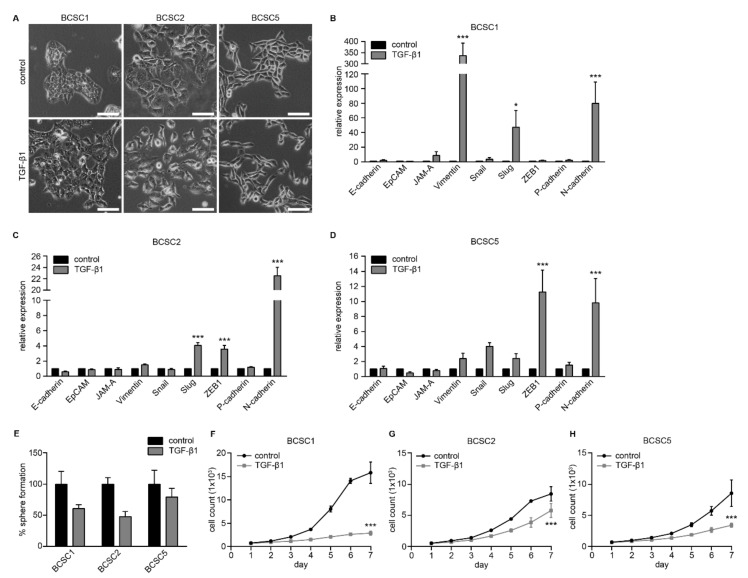
TGF-β1 promotes mesenchymal features in BCSCs. (**A**) Morphology of BCSC1, BCSC2, and BCSC5 without or with TGF-β1 stimulation. Cell morphology was documented using phase contrast microscopy. Scale bars represent 100 μm. (**B**–**D**) Analysis of EMT-related gene expression in BCSC1 (**B**), BCSC2 (**C**), and BCSC5 (**D**) without or with TGF-β1 stimulation using qRT-PCR (*n* = 3). Data represents means + SEM. * *p* < 0.05, *** *p* < 0.001 by two-way ANOVA. (**E**–**H**) Growth of BCSC1, BCSC2, and BCSC5 without or with TGF-β1 stimulation. Sphere formation in 50% Matrigel was analyzed relative to control cells (**E**). Proliferation of BCSC1 (**F**), BCSC2 (**G**), and BCSC5 (**H**) was assessed every day for 7 days by counting DAPI-positive nuclei (*n* = 2). Data represents means ± SEM. *** *p* < 0.001 by two-way ANOVA.

Indeed, treatment with 5 ng/mL TGF-β1 daily for 10 days altered BCSC morphology significantly (Figure 4A). For BCSC1, cell–cell contacts were reduced, and cell size increased compared to untreated control cells, which formed well-defined epithelial colonies with cobblestone morphology. For BCSC2, the formation of loose cell clusters was observed compared to the epithelial colonies of untreated control cells. BCSC5 cell morphology was more elongated, and the colonies lost the cobblestone morphology which was characteristic for the control cells.

The shift to a more mesenchymal phenotype was also reflected in the EMT-related gene expression profiles of BCSC1, BCSC2, and BCSC5 analyzed after 10 days of treatment with TGF-β1. BCSC1 significantly upregulated mesenchymal genes such as vimentin, Slug, and N-cadherin compared to the control cells (Figure 4B). For BCSC2, a significant upregulation of Slug, ZEB1, and N-cadherin was documented (Figure 4C). BCSC5 significantly upregulated ZEB1 and N-cadherin and showed a tendency to upregulate vimentin, Snail, and Slug (Figure 4D). The gene expression alterations after TGF-β1 stimulation observed at the mRNA level were further confirmed at the protein level using Western blot analysis (Figure S8) and immunocytochemistry (Figure S9).

TGF-β1 has been described to have a dual function in cancer as both tumor suppressor and promotor [40]. In accordance with studies suggesting that TGF-β1 can act as a tumor suppressor [41], BCSCs formed fewer spheres in 3D sphere formation assays using 50% Matrigel upon TGF-β1 treatment (Figure 4E). Furthermore, proliferation was impaired compared to untreated control cells in 2D assays using 2% Matrigel (Figure 4F–H).

**Figure 5:**
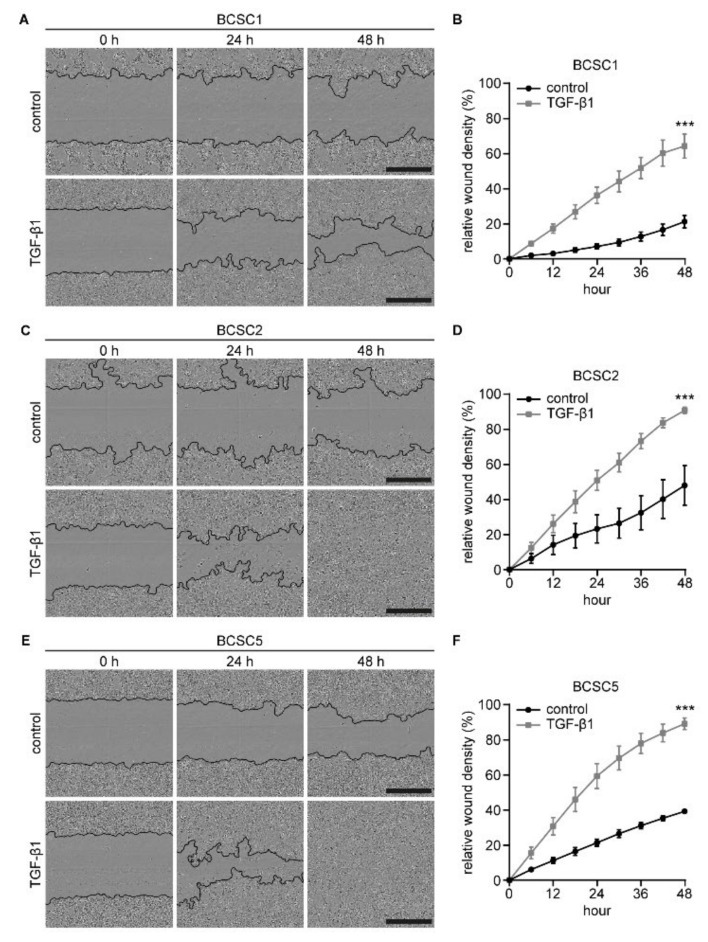
Stimulation with TGF-β1 promotes BCSC migration. Scratch wound assays with and without TGF-β1 stimulation monitored over 48 h. (**A**,**C**,**E**) Depicted are representative images at 0 h, 24 h, and 48 h for BCSC1 (**A**), BCSC2 (**C**), and BCSC5 (**E**). Detected wound edges are marked by a black line. Scale bars represent 500 μm. (**B**,**D**,**F**) Quantification of wound closure is depicted in relative wound density in percent (*n* = 2). Data represents means ± SEM. *** *p* < 0.001 by two-way ANOVA.

TGF-β1 has also been reported to promote tumor progression by enhancing migration and invasion [40,41]. To analyze the effect of TGF-β1 stimulation on BCSC migration, BCSCs were treated for 5 days prior to seeding into 96-well plates. Cells were cultured and treated for a further 48 h to allow confluency before scratch wounds were inflicted. Wound closure was monitored for 48 h and documented as described above. For all three cell lines, the migration capacity was enhanced significantly (Figure 5). After 48 h, a final mean RWD of 21.2% was documented for BCSC1 control cells whereas TGF-β1-treated cells reached a final mean RWD of 64.3% (Figure 5A,B). For BCSC2 control cells, a final mean RWD of 48.0% was documented. TGF-β1 treatment enhanced the final mean RWD to 90.8% (Figure 5C,D). BCSC5 control cells reached a final mean RWD of 39.2%, whereas for TGF-β1-treated cells, a final mean RWD of 89.3% was observed (Figure 5E,F).

Taken together, we could show that stimulation with TGF-β1 induced morphological changes and upregulation of mesenchymal markers in BCSCs. Furthermore, even though sphere formation and proliferation were reduced upon stimulation with TGF-β1, migratory abilities of BCSCs were enhanced. This highlights the dynamic state of BCSCs, suggesting that they can respond flexibly to cues from the tumor microenvironment.

## 3. Discussion

For breast cancer and other cancers, it has been suggested that a small population of CSCs in the tumor drive tumor progression and potentially metastasis formation. CSCs are hypothesized to have profound implications for cancer therapy since they are difficult to treat with conventional therapies [6,42], making investigation of this cell type particularly important. Due to the rarity of stable BCSCs within cancer cell populations in vitro, there is a need for new reliable cell culture models that are enriched for BCSCs and are suitable tools for both in vitro and in vivo studies. Utilizing our previously published isolation method [31], here we present and characterize two new BCSC lines that were derived from TNBC patient tumors.

BCSC3 and BCSC4 form tumor spheres when cultivated in a hypoxic 3D environment and grow as adherent cell colonies when grown under 2D culture conditions. Their BCSC potential was evaluated by anchorage-independent growth in vitro and further confirmed by transplantation of low cell numbers into mice where reliable tumor formation was observed. Importantly, the xenograft tumors phenocopied the original patient tumors in morphology, tumor cell architecture, breast cancer subtype, and gene expression.

Utilizing these promising models, we investigated various characteristics of triple-negative BCSCs. The cells exhibited a bipotent precursor phenotype by co-expressing myoepithelial and luminal epithelial markers. They also concomitantly expressed epithelial and mesenchymal markers, suggestive of a state of epithelial-mesenchymal plasticity. This plasticity was further confirmed when the cells were exposed to TGF-β1, resulting in a shift to a more migratory, mesenchymal phenotype.

The BCSC lines described here together with the ones previously published by us [31,32] present promising tools to study BCSC characteristics because, compared to conventional cell lines, which are the most frequently used models in breast cancer research [26], they are enriched for BCSCs.

Anchorage-independent sphere formation, which is a common approach to evaluate cancer stem cell potential in vitro [28,43], was higher in the BCSC lines than in various other published studies that used conventional cell lines. There, sphere formation ranged from 0.5 to 5.5% [28,44,45,46,47], whereas BCSC3 and BCSC4 showed a capacity for sphere formation of approximately 13%. When their CSC potential was tested in vivo through orthotopic transplantation into mice, both BCSC3 and BCSC4 proved highly tumorigenic. BCSC3 formed tumors when as few as 1 × 10^3^ cells were transplanted. BCSC4 started to reliably form tumors at 1 × 10^4^ transplanted cells. Transplantation of conventional breast cancer cell lines such as MDA-MB-231 and MCF7 often requires at least 1 × 10^5^ to 1 × 10^6^ cells to form tumors [27,28], potentially due to low numbers of cancer stem cells in the cultures [29,30]. Still, even when conventional cell cultures were enriched for BCSCs using designated BCSC surface markers, at least 2 × 10^5^ cells were required to form tumors in mice [6]. This emphasizes the high intrinsic percentage of tumorigenic cells in the BCSC lines presented here.

Additionally, it has been criticized that conventional cell lines tend to lose clonality over time, resulting in phenotypically homogeneous cell cultures [48]. The primary BCSC cultures described here exhibited stable intracultural heterogeneity over all monitored passages.

Most importantly, it is hypothesized that CSCs have the ability to form tumors that recapitulate the heterogeneity of the tumors they were isolated from [49]. This was confirmed by BCSC3 and BCSC4 phenocopying the original patient tumors in their morphology and gene expression. The xenograft tumor and the corresponding patient tumor showed a similar mixed epithelial and mesenchymal phenotype, as well as comparable keratin patterns. Markers relevant for receptor status were similarly expressed, resulting in the same clinicopathological classification, i.e., the triple-negative subtype. This histopathological resemblance was previously further confirmed by us at the transcriptional level at a genome-wide scale using RNA microarray experiments [31]. Therefore, xenografts from BCSCs closely mimic the patient situation and represent promising preclinical models, for example for personalized medicine.

Since BCSCs are thought to present a minor population within the tumor and corresponding tumor cell cultures [11], it is essential to identify markers to distinguish BCSCs from the non-tumorigenic cancer cells in the population. Different marker combinations have been proposed for this purpose, including CD44^+^/CD24^−^ [15]. Both BCSC lines described here differed in their expression of CD44 and CD24. Interestingly, the marker expression did not correlate completely with the functional BCSC properties observed. The higher proportion of CD44^+^/CD24^−^ cells in BCSC4 would suggest a higher BCSC potential, but BCSC4 showed less colony and sphere formation and substantially slower tumor growth compared to BCSC3.

This inconsistency between the CD44^+^/CD24^−^ phenotype and the correlated tumorigenicity has already been documented for other breast cancer cells cultivated as adherent colonies or mammospheres [50,51] and for the TNBC cell line MDA-MB-468 which consists mainly of CD44^+^/CD24^+^ cells despite being tumorigenic [52], similarly to BCSC3. This suggests that the hypothesized correlation between CD24 negativity and tumorigenicity is not always applicable and that a universal molecular marker remains to be identified that recognizes BCSCs exclusively.

Other studies suggest expression of EpCAM and CD49f as BCSC markers. EpCAM has been reported to be expressed in luminal cells, whereas high expression of CD49f marks basal cells [53]. Human bipotent mammary progenitor cells are described to express both markers simultaneously [54]. Analyses of BCSC3 and BCSC4 showed that CD49f was expressed by all cells of both BCSC lines while EpCAM expression varied slightly. However, the majority of cells were positive for both markers, indicating a bipotent precursor phenotype for triple-negative BCSCs.

This observation was further corroborated by the co-expression of myoepithelial and luminal epithelial keratins in all BCSC lines investigated. This is in accordance with studies suggesting that triple-negative breast tumors often show a bipotent progenitor phenotype with poor histopathological differentiation [55,56,57]. Therefore, it remains elusive if the documented bipotent phenotype of the BCSCs results from their origin in triple-negative breast cancer tissue or if it can be attributed to their nature as cancer stem cells.

Several studies suggest that even though cancer stem cells are not necessarily derived from normal stem cells, they share functional similarities with them [12,58]. There is further evidence that expression of certain transcription factors can reprogram normal differentiated cells to induced pluripotent stem cells [59], and it is thought that these transcription factors could be involved in CSC formation and maintenance [60]. Therefore, we analyzed the expression of embryonic stem cell transcription factors at the transcriptional level in all BCSC lines, however, no expression of Sox2 and Nanog, and only very low expression of Oct4, was detected. This is in contrast to studies that have previously linked all three transcription factors to breast cancer [61,62,63,64]. Compared to human embryonic stem cells, our BCSCs express these stem cell-related transcription factors at barely detectable levels. That is why we did not focus further on functional correlations in the current study.

Overall, defining suitable describing marker profiles for BCSCs poses a considerable challenge given the vast phenotypic heterogeneity found in breast cancer tumors, even of the same histopathological subtype, and more studies will be required to achieve this goal. Due to the presented heterogeneity in marker expression between the different BCSC lines, but also within different cell populations of the same BCSC line, the cells presented here can be used to address this goal, for example by sorting for specific marker expression [65]. Subsequent transcriptomic or proteomic in-depth analysis could then be applied to define other commonly expressed genes of interest in the different populations, which could be used as future biomarkers of BCSCs [66].

In recent years, different studies have hypothesized that the cancer stem cell state is linked to the EMT state of the cell, and that BCSCs can exist in different EMT states [19,20]. That is why we analyzed the primary BCSC lines with regard to their EMT marker profile. BCSCs showed concomitant expression of different epithelial and mesenchymal markers such as keratins and vimentin, respectively, but also simultaneous expression of E-cadherin and several EMT-related transcription factors. This is in agreement with studies hypothesizing that BCSCs can exist both in a more mesenchymal-like and in a more epithelial-like state, but that BCSCs with both characteristics show the greatest tumor-initiating capacity [16,21,25]. Interestingly, although general co-expression of epithelial and mesenchymal markers was detected for all BCSCs, the expression of specific markers varied between individual BCSC lines. This suggests that even though the EMP state was observed for all BCSC lines, the molecular drivers may vary between the BCSC lines emphasizing their individuality.

The observed EMP state has been suggested to provide the BCSCs with the flexibility to fulfil the diverse requirements occurring during tumor progression [23]. One of the requirements BCSCs are confronted with within the tumor is their adaptation to soluble stimuli present in the tumor microenvironment such as the cytokine TGF-β1. Stimulation of BCSC1, BCSC2, and BCSC5 with TGF-β1 resulted in morphological alterations such as a reduction of the cobblestone-like morphology as well as cell–cell contacts and the occurrence of a more elongated cell shape. Furthermore, the EMT marker profile was altered, showing a reduction in epithelial marker expression and an increase in mesenchymal marker expression. This is in accordance with an induced EMT phenotype. Interestingly, only a limited number of TGF-β-inducible human breast cancer cell culture models is available [67], and none of them are characterized as primary cell lines enriched for CSCs, suggesting the BCSC lines presented here as a promising in vitro tool to analyze induced EMT in BCSCs.

Furthermore, TGF-β1-treated BCSCs showed decreased anchorage-dependent sphere formation in 3D cultures and reduced cell proliferation in 2D cultures. This is in accordance with the hypothesis that TGF-β1 acts as a tumor suppressor by inhibiting cancer cell growth [40,41]. At the same time, it is known that TGF-β1 can act as a tumor promotor by enhancing migratory and metastatic capacities of cancer cells. This was reflected in the significantly enhanced migratory abilities of TGF-β1-treated BCSCs in comparison to untreated control cells which showed only limited migratory potential. These observations highlight the plasticity of BCSCs which facilitates their responsiveness to microenvironmental cues. Without the external stimulation, the BCSCs analyzed here showed a highly proliferative, epithelial phenotype with limited migratory capacity. However, when they were confronted with TGF-β1, their self-renewal was decreased and their migratory abilities increased, indicating a shift to a more mesenchymal phenotype. We hypothesize that the observed plasticity provides the BCSCs with the needed flexibility to maintain tumor growth throughout the different stages of tumor progression.

## 4. Materials and Methods

### 4.1. Tissue Specimens

Tissue specimens of human primary breast cancer tumors were obtained from pathologists of the tumor bank of the Comprehensive Cancer Centre Freiburg after operation at the Department of Obstetrics and Gynecology at the University Medical Center Freiburg. Specimens were used for isolation of breast cancer stem cells and for paraffin embedding after fixation.

### 4.2. BCSC Culture

BCSC3 and BCSC4 were isolated from primary triple-negative breast cancer tissue of patients using the same methodology as described in our previous publication [31].

All BCSC lines were cultured in MSC medium consisting of MEBM (Lonza, Basel, Switzerland) supplemented with 2% B27 (Thermo Fisher Scientific, Waltham, MA, USA), 1% Amphotericin B (Sigma-Aldrich, St. Louis, MO, USA), 1% Penicillin-Streptomycin (Gibco), 20 ng/mL EGF (PeproTech, Rocky Hill, NJ, USA), 4 μg/mL heparin (Sigma-Aldrich), 20 ng/mL FGF (PeproTech), 35 μg/mL gentamicin (Thermo Fisher Scientific), 500 nM H-1152 (Calbiochem, Sigma-Aldrich, St. Louis, MO, USA) at 37 °C and under low-oxygen conditions (3% O_2_, 5% CO_2_, 92% N_2_).

Initial 3D cultures were grown in 50% Matrigel. Upon stable proliferation in 3D cultures, cells were cultured and expanded in a 2D environment using 2% Matrigel. To subculture spheres grown in a 3D environment, BCSC spheres were dissociated from residual Matrigel with Dispase (Corning Inc., Corning, NY, USA). They were resuspended in Accutase (Sigma-Aldrich) and incubated at 37 °C for 20 min to obtain a single cell suspension. To subculture cells grown in a 2D environment, BCSC cultures were incubated with Accutase at 37 °C for 15 to 30 min to obtain a single cell suspension. In both cases, the cell numbers were determined, and a suitable cell number was reseeded into a new vessel. Subculturing of 3D cultures was performed every week for BCSC1, BCSC2, and BCSC5, and every other week for BCSC3 and BCSC4. Subculturing of 2D cultures was performed once per week for all BCSCs.

Documentation of cells was performed using the DM IL LED microscope by Leica.

All experiments were performed with BCSCs cultured in 2D on 2% Matrigel if not indicated otherwise. For all conducted experiments, BCSCs with a passage number lower than 35 were used.

### 4.3. Conventional Cell Culture

MCF7 and MDA-MB-231 were cultured in DMEM medium (Thermo Fisher Scientific) supplemented with 10% FBS (Thermo Fisher Scientific) and 1% Penicillin-Streptomycin (Thermo Fisher Scientific) at 37 °C and under 5% CO_2_. For subculturing, the cells were detached with Trypsin (Thermo Fisher Scientific), and a suitable amount of cell suspension was reseeded into a new culturing flask. MCF7 and MDA-MB-231 were subcultured twice per week with approximate rates of 1:6 and 1:12, respectively.

### 4.4. Cell Proliferation Assay

To monitor cell proliferation, 384-well μ-clear plates (Greiner, Kremsmuenster, Austria) were coated with 10 μL of MSC medium containing 2% Matrigel. After solidification of the Matrigel at 37 °C for 30 min, 0.5 × 10^3^ cells in 70 μL per well were seeded in six technical replicates. To observe growth over 7 days, 7 individual plates were generated, and one plate was fixed every 24 h with methanol at −20 °C. Subsequently, cell nuclei were stained with DAPI staining buffer (10 mM Trishydrochloride, 10 mM EDTA, 100 mM NaCl, 0.3 μg/mL DAPI), and the cells present in the entire well were counted using the Olympus ScanR microscope, using the 10× NA0.4 UPLSAPO objective and ScanR analysis software v2.6.2.

### 4.5. Colony Formation Assay

12-well plates were coated with 200 μL of MSC medium containing 2% Matrigel. After solidification of the Matrigel at 37 °C for 30 min, 0.2 × 10^3^ single cells per well were seeded in 800 μL MSC medium. Cells were grown at 37 °C and under low-oxygen conditions for 8 days. After that, cells were fixed with methanol at −20 °C and stained with 0.05% crystal violet dissolved in H_2_O_dest_. for 3 min. After washing with H_2_O_dest_, the plates were air-dried, and colonies were counted using a stereo microscope. Cell clusters larger than 8 cells were counted as colonies.

### 4.6. Sphere Assay in Matrigel

For quantification of anchorage-dependent sphere-forming capacity, 0.5 × 10^3^ single cells were seeded into 24-well ultralow attachment plates (Corning Inc.) in a 1:1 mixture of Matrigel and MSC medium and cultured as described above. After 8 days, spheres with a diameter of over 50 μm were counted using a stereo microscope.

### 4.7. Sphere Assay in Methylcellulose

For quantification of anchorage-independent sphere-forming capacity, 0.5 × 10^3^ single cells were seeded into 96-well ultralow attachment plates in MSC medium containing 1% methylcellulose (Sigma-Aldrich). Cells were cultured as described above. After 10 days, spheres with a diameter of over 50 μm were counted using a cell culture microscope.

### 4.8. Immunofluorescence Staining

To analyze intracellular protein localization, 96-well μ-clear plates (Greiner) were coated with MSC medium containing 2% Matrigel as described above. 3 × 10^3^ to 12 × 10^3^ cells per well were seeded and grown for 3 to 6 days. Subsequently, they were fixed either with ice-cold methanol for at least 30 min at −20 °C or with 4% PFA for 20 min on ice.

Cells were washed with DPBS and permeabilized with TBST for 6 min at room temperature. After blocking with 1 mg/mL ovalbumin (Sigma-Aldrich) in DPBS (ova/DPBS) for 1 h at room temperature, cells were incubated with primary antibody diluted in ova/DPBS over night at 4 °C (Table S1). The following day, cells were washed with DPBS and incubated with fluorescently labeled secondary antibody diluted in ova/DPBS for 1 h at room temperature (Table S1). Finally, after washing with DPBS, the nuclei were stained with DAPI staining buffer. Images were taken using the fluorescence unit of the Zeiss AxioObserver ApoTome.v2, using the 10x NA 0.3 EC Plan-Neofluar objective and analyzed using Zeiss Zen v2.

### 4.9. Flow Cytometry

To analyze the expression of established BCSC markers, cells were detached and counted as described above. Approximately 1 × 10^5^ cells were washed with staining buffer (DPBS containing 1% BSA) and stained for 20 min at room temperature in the dark with the antibodies of interest diluted in staining buffer (Table S1). Cells were analyzed using a BD LSR Fortessa and FlowJo software v7 (FlowJo, LLC, Ashland, OR, USA).

### 4.10. Orthotopic Breast Cancer Xenografts

Surgeries were performed as previously published [31]. Animals were monitored twice per week for animal weight and tumor growth, which was determined by caliper measurement. Tumor volumes were calculated using the formula 4/3 · π · r^3^.

Limiting dilutions were calculated using the Extreme Limiting Dilution Analysis (ELDA) software [33].

### 4.11. Immunohistochemistry

Immunohistochemical analysis was performed as previously described [31]. As internal positive controls, a patient-derived physiological mammary gland was used for ER and PR staining. For HER2, tissue specimens from HER2-positive breast cancer patients (score 3) were included. Triple-negative breast cancer was defined as ER-, PR-, and HER2-negative (score < 2) breast cancer [2]. The Ki67-labeling index was conventionally determined by counting Ki67-positive nuclei in tissue sections and scored as low, intermediate, and high if less than 15%, between 16% and 30%, and more than 30%, respectively, of cell nuclei were positive [68].

### 4.12. Quantitative Real-Time PCR Analysis

To purify total RNA, cells were cultured as described above and subsequently lysed in RLT Buffer of the RNeasy Mini Kit (Qiagen, Venlo, Netherlands). RNA purification was performed according to the manufacturer’s instructions and total RNA concentration was determined using a NanoDrop 2000. Total RNA was stored at −80 °C.

For further analyses, total RNA was transcribed into complementary DNA (cDNA) using the RevertAid First Strand cDNA Synthesis Kit (Thermo Fisher Scientific) according to the manufacturer’s instructions. Finally, cDNA was diluted to 3 ng/μL in RNase-free water and stored at −20 °C.

For quantitative real-time polymerase chain reaction (qRT-PCR), 7.5 ng of cDNA were added to a qRT-PCR reaction mix containing TaqMan Universal Master Mix II (Thermo Fisher Scientific), forward and reverse primers (Table S2), and the corresponding Universal Probe Library (UPL) probe (Roche, Basel, Switzerland). Amplification and analysis was carried out using a LightCycler 480 (Roche). The following thermocycler settings were used: Activation, 95 °C, 10 min; 50× amplification, 95 °C, 15 s, 60 °C, 1 min; cooling, 4 °C.

### 4.13. Western Blot Analysis

To isolate whole cell protein, cells were cultured as described above. Subsequently, they were washed with DPBS, scraped off, and collected via centrifugation at 200 g for 3 min. The cell pellet was resuspended in an appropriate volume of triple detergent lysis buffer containing PMSF (Thermo Fisher Scientific) and cOmplete™ Protease Inhibitor Cocktail (Sigma-Aldrich) and incubated for 30 min on ice. To remove cell debris, lysates were centrifuged at 13,000 rpm for 15 min at 4 °C and the protein-containing supernatant was transferred to a new reaction tube.

Protein concentrations were determined using the DC™ Protein Assay Kit II (Bio-Rad Laboratories, Hercules, CA, USA) according to the manufacturer’s instructions and the Spark^®^ 10M Multimode Microplate Reader measuring absorbance at 750 nm. As a point of reference, a BSA serial dilution was analyzed in parallel to generate a standard curve.

For SDS-polyacrylamide gel electrophoresis, 30 μg of protein lysate was filled up to 15 μL with DPBS and mixed with 4× loading buffer. The mixture was heated to 95 °C for 5 min and loaded on a polyacrylamide gel. PageRuler Prestained Protein Ladder (Thermo Fisher Scientific) was loaded as reference.

After SDS-PAGE, blotting was performed at 300 mA for 2.5 h using a wet blotting system by Bio-Rad Laboratories. Afterwards, the membrane was blocked with 5% milk powder in TBST for 1 h. Indicated antibodies (Table S1) were diluted in blocking solution and incubated over night at 4 °C. The following day, membranes were washed with TBST, followed by incubation with the respective HRP-conjugated secondary antibody diluted in 5% milk powder for 1 h at room temperature. After washing with TBST, the membrane was incubated with Amersham ECL Prime Western Blotting Detection Reagent (GE Healthcare, Chicago, IL, USA) for 3 min, and chemiluminescent signal was detected using a Universal Hood III by Bio-Rad Laboratories. Uncropped Western blots are depicted in Figure S10. Densitometry readings/intensity ratios relative to β-actin are shown in Figure S11.

### 4.14. Scratch Wound Assay

To analyze cell migration, cells were seeded in 96-well plates at a density that reached confluence after 2 days of culture. One 700 to 800 μm wide scratch wound per well was inflicted using the IncuCyte WoundMaker (Sartorius, Goettingen, Germany) according to the manufacturer’s instructions. Scratch wound closure was subsequently monitored for 48 h by taking images every hour using the IncuCyte S3 Live-Cell Analysis System (Sartorius).

Quantifications are depicted as relative wound density (RWD) in percent. RWD measures the spatial cell density inside the wound area relative to the spatial cell density outside the wound area at every point in time, being 0% when the wound is inflicted and reaching 100% when the cell density inside the wound is the same as the cell density outside the initial wound.

### 4.15. Stimulation with TGF-β1

TGF-β1 (PeproTech) was dissolved in citric acid (10 mM, pH 3) and diluted in DPBS containing 1% BSA to a final stock concentration of 20 μg/mL. Aliquots were stored at −20 °C. For stimulation of cells, a final concentration of 5 ng/mL TGF-β1 in MSC medium was used, and TGF-β1 was added daily. Cells were pre-treated for 5 days prior to the experiments, and the treatment was continued over the duration of the experiment.

### 4.16. Statistics

Statistical analysis was performed using GraphPad Prism v8. Data represents means ± SEM unless otherwise indicated. Not significant (ns), *p* > 0.05; *, *p* ≤ 0.05, **, *p* ≤ 0.01; ***, *p* ≤ 0.001.

## 5. Conclusions

BCSCs represent a particularly important cell population within the tumor due to their self-renewal abilities and their difficulty to treat with conventional therapies. Therefore, investigation of BCSCs is crucial and needs reliable models for in vitro and in vivo studies. Here, we presented newly established BCSC lines from TNBC and validated their BCSC characteristics in vitro and in vivo. Importantly, when xenografted into mice, the BCSC lines can phenocopy the original patient tumor in its architecture and gene expression. These models were subsequently used to analyze the EMT state of triple-negative BCSCs. We found concomitant expression of epithelial and mesenchymal markers, hinting at the highly flexible state of epithelial-mesenchymal plasticity. This plasticity was confirmed by stimulating the cells with TGF-β1, which resulted in an upregulation of mesenchymal features. We therefore conclude that BCSCs can react flexibly to microenvironmental cues in the tumor. Overall, the primary BCSC lines we present here are a valuable tool to address questions of BCSC biology and to evaluate new treatment options for TNBC.

## Data Availability

The data presented in this study are available on request from the corresponding author.

## Supplementary Materials

The following are available online at https://www.mdpi.com/1422-0067/22/4/1808/s1, Figure S1: BCSC patient and xenograft tumor tissues are classified as triple-negative, Figure S2: BCSCs exhibit morphological heterogeneity in vitro, Figure S3: BCSCs express myoepithelial keratin 14 and luminal epithelial keratin 18, Figure S4: Expression of stem cell-related transcription factors in BCSCs, Figure S5: BCSCs co-express E-cadherin and vimentin, Figure S6: BCSCs differ in their migratory potential, Figure S7: BCSCs express genes related to the TGF-β signaling pathway, Figure S8: Expression of EMT-related markers on the protein level after TGF-β1 stimulation, Figure S9: E-cadherin and vimentin expression of BCSCs after TGF-β1 stimulation, Figure S10: Uncropped Western blots, Figure S11: Densitometry readings/intensity ratios relative to β-actin, Figure S12: BCSCs co-express epithelial and mesenchymal markers in 3D cultures, Table S1: List of antibodies, Table S2: Oligonucleotides for qRT-PCR.
