# Supplementary material for: Human Primary Breast Cancer Stem Cells Are Characterized by Epithelial-Mesenchymal Plasticity
Source: Int J Mol Sci. 2021 Feb 11;22(4):1808. doi: 10.3390/ijms22041808 (PMC7918351; doi:10.3390/ijms22041808)
Supplement: Supplementary file 1 [file ijms-22-01808-s001.pdf]

International Journal of
Molecular Sciences

MDPI

Supplementary Materials

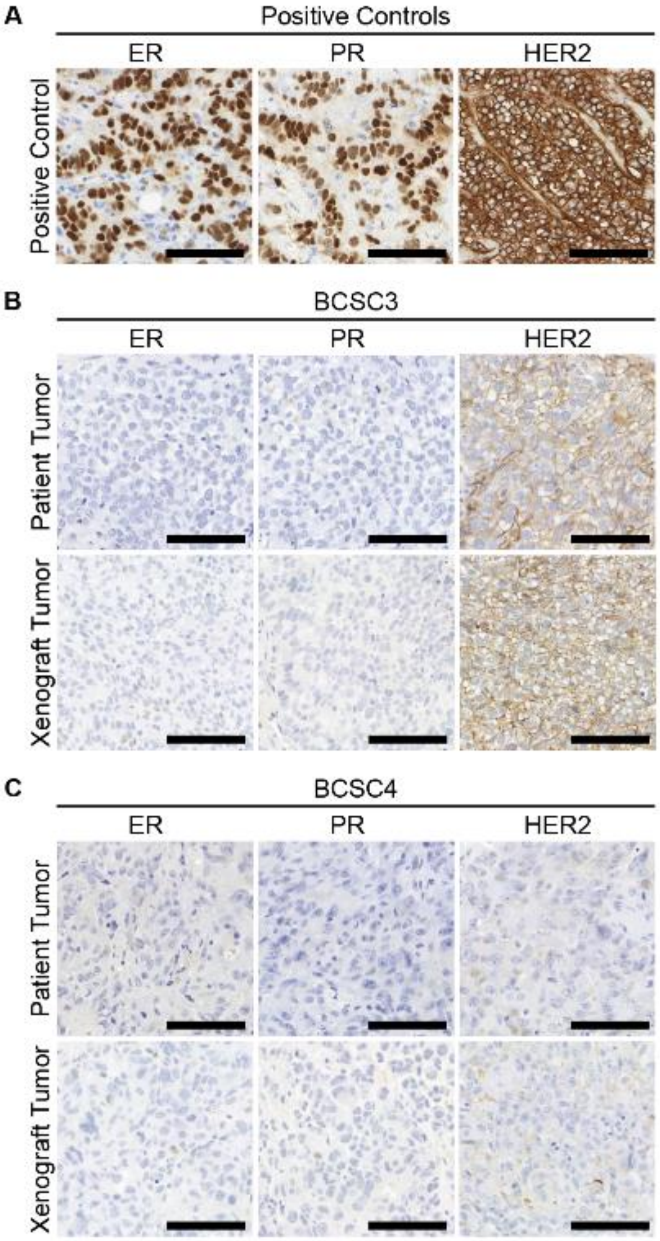

Figure S1. BCSC patient and xenograft tumor tissues are classified as triple-negative. (**A**) Representative images of positive controls for ER, PR, and HER2 expression. (**B**, **C**) Patient and xenograft tumor tissue of BCSC3 (**B**) and BCSC4 (**C**) were fixed, paraffin-embedded, and sectioned. Depicted are representative images of immunohistochemical analysis of ER, PR, and HER2 expression. Scale bars represent 100 µm.

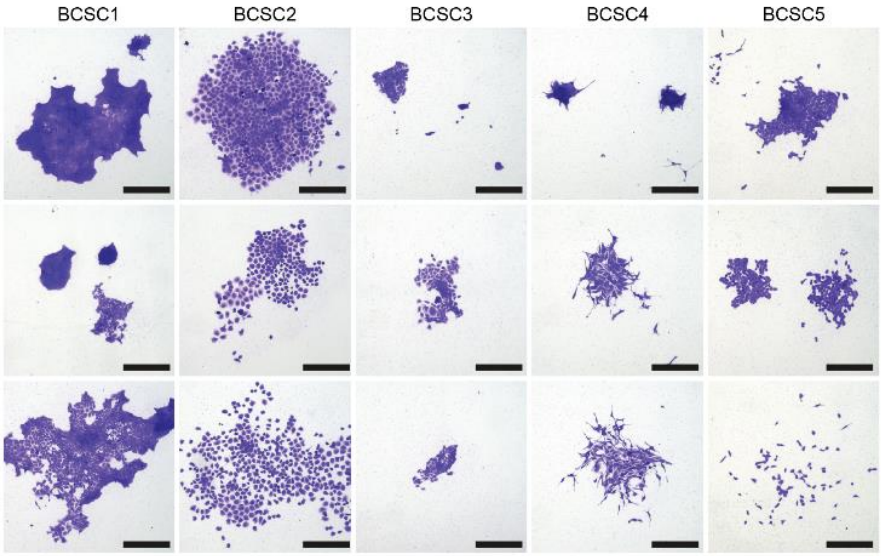

Figure S2. BCSCs exhibit morphological heterogeneity in vitro. BCSCs were seeded as single cells in a 2D environment and grown for 8 days. Cells were fixed and stained with crystal violet to enable better observation of the colony morphologies. For each BCSC line three exemplary colony morphologies are depicted. Scale bars represent 400 µm.

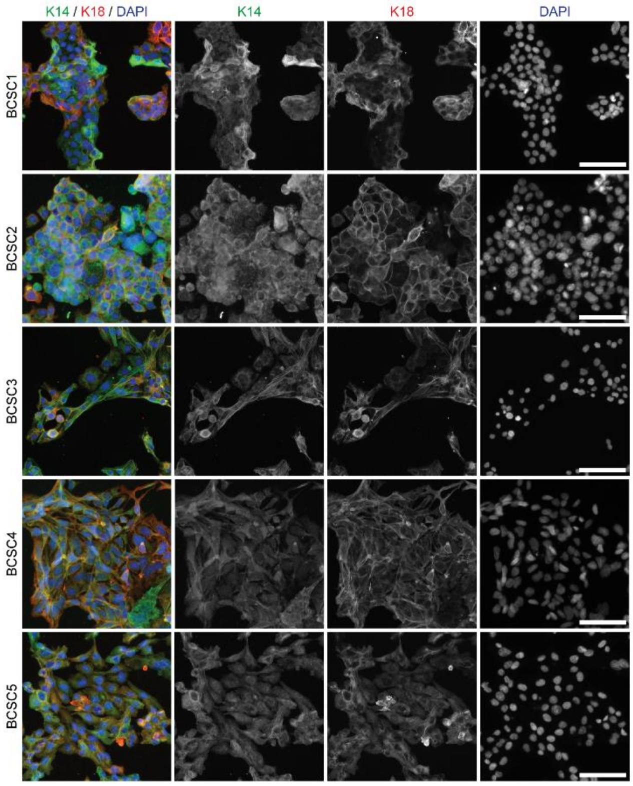

Figure S3. BCSCs express myoepithelial keratin 14 and luminal epithelial keratin 18. Representative images depicting immunofluorescence staining of BCSCs using antibodies against K14 and K18. Nuclei were counterstained with DAPI. Merge and single channels of extended depth of focus z-stack images are shown. Scale bars represent 100 µm.

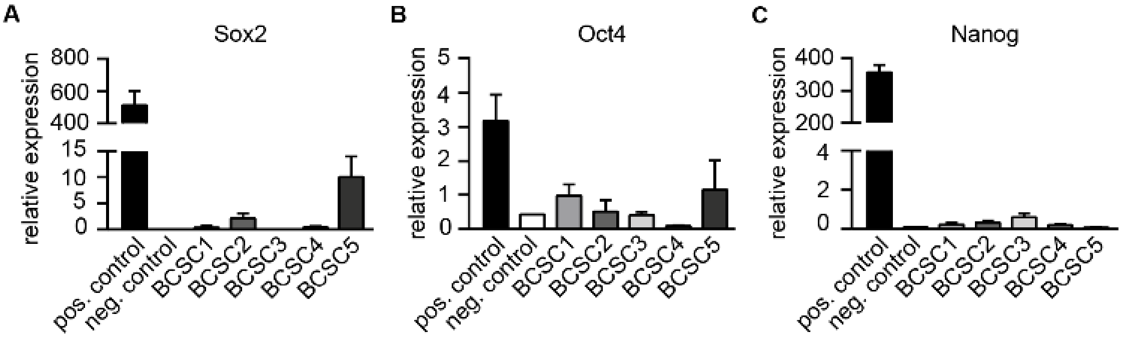

Figure S4. Expression of stem cell-related transcription factors in BCSCs**.** Analysis of Sox2 (**A**), Oct4 (**B**) and Nanog (**C**) mRNA expression in BCSCs using qRT-PCR. Depicted mRNA levels are relative to the housekeeping gene HPRT1 (n=3). Data represents means + SEM.

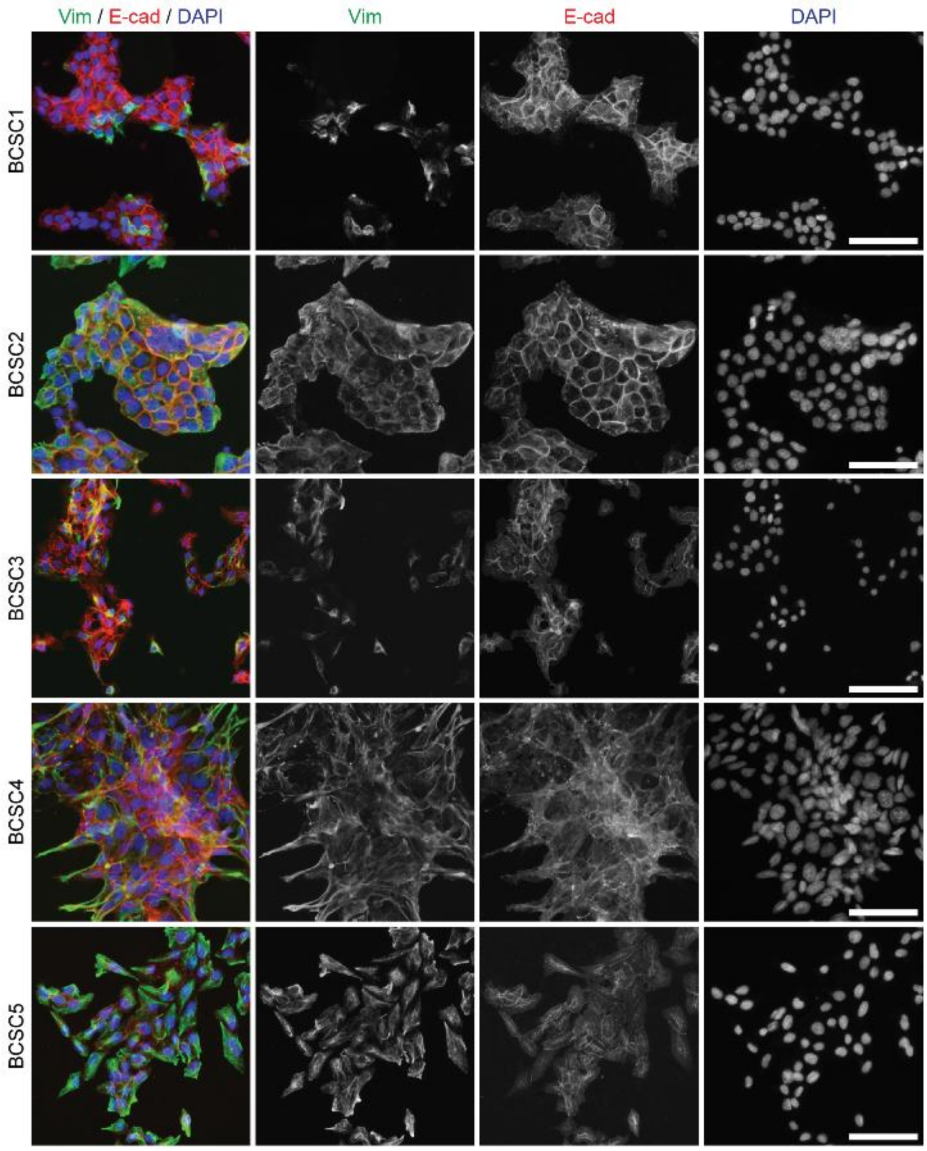

Figure S5. BCSCs co-express E-cadherin and vimentin. Single channels of immunofluorescence analysis of BCSCs shown in Figure 3A. Antibodies against epithelial E-cadherin (red) and mesenchymal vimentin (green) were used. Cell nuclei were counterstained with DAPI. Scale bars represent 100 µm.

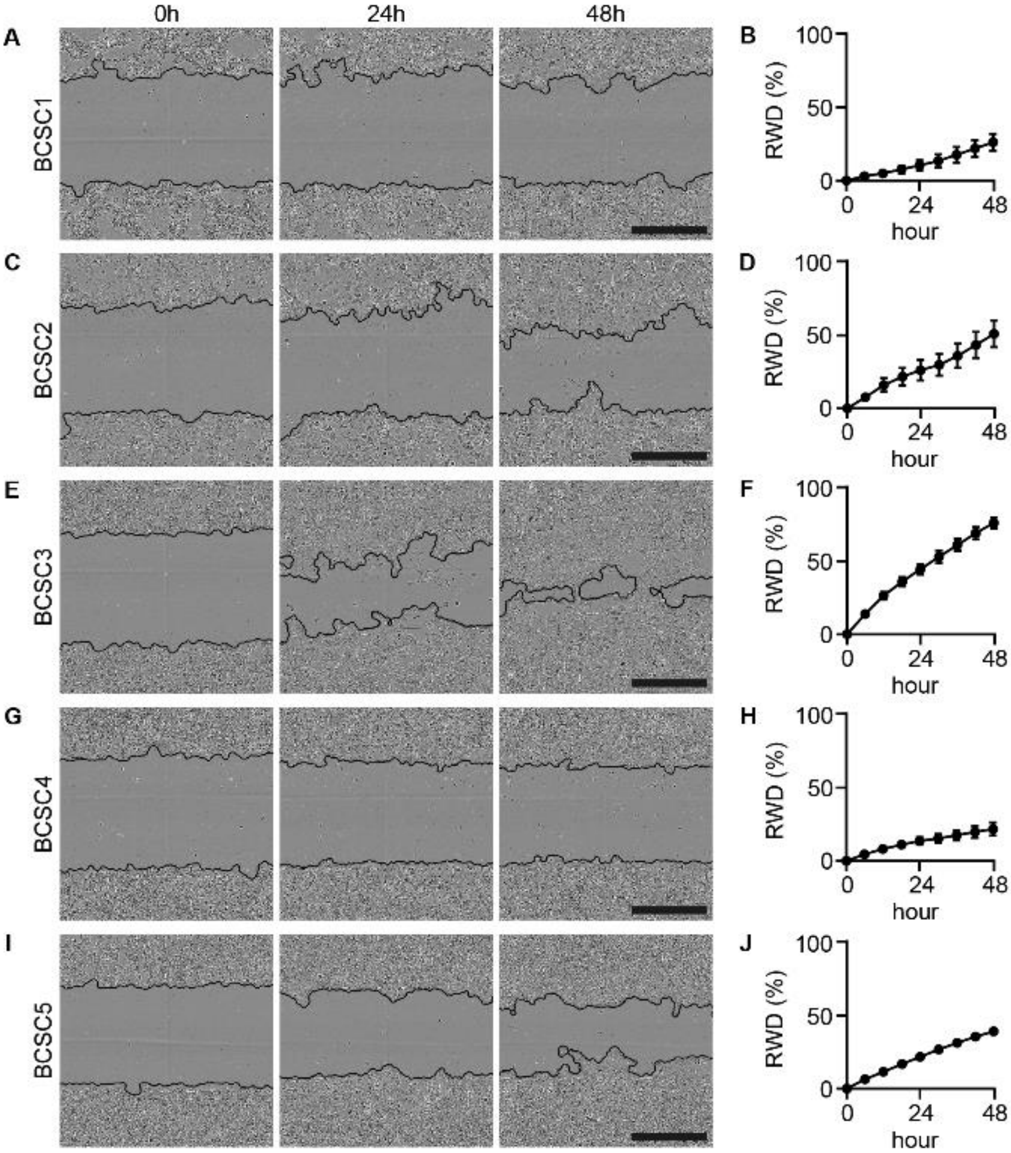

Figure S6. BCSCs differ in their migratory potential. BCSCs were grown to 100% confluency, scratch wounds were inflicted, and cell migration was monitored over 48 h. (**A**, **C**, **E**, **G**, **I**) Depicted are representative images at 0 h, 24 h and 48 h for each cell line. Detected wound edges are marked in each case by a black line. Scale bars represent 500 µm. (**B**, **D**, **F**, **H**, **J**) Quantification of wound closure is depicted in relative wound density (RWD) in percent (n=2). Data represents means ± SEM.

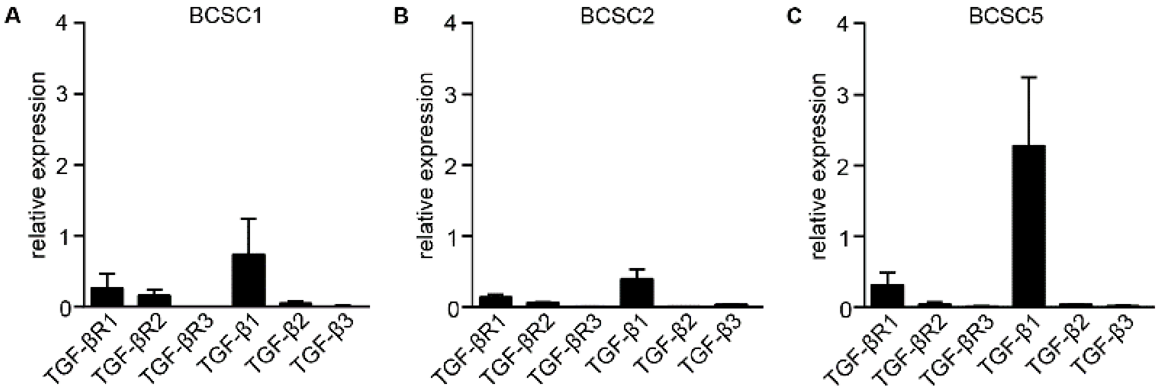

Figure S7. BCSCs express genes related to the TGF-β signaling pathway. (**A**-**C**) Analysis of TGF-β-related receptors and ligands analyzed by qRT-PCR. Values are mRNA levels relative to HPRT1 (n=3). Data represents means + SEM.

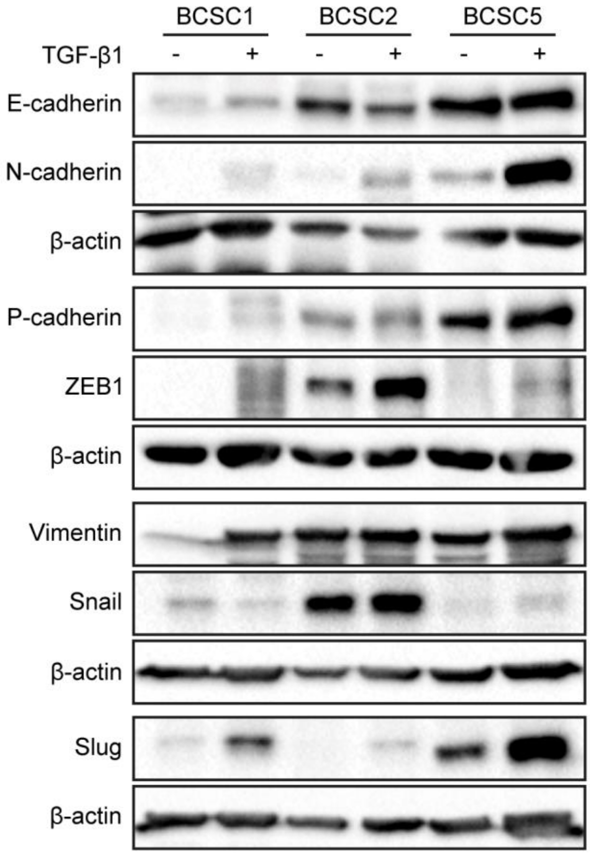

Figure S8. Expression of EMT-related markers at the protein level after TGF-β1 stimulation. Expression of different EMT-related proteins was analyzed by SDS-PAGE and Western blot in BCSC1, BCSC2, and BCSC5 with and without TGF-β1 stimulation (n=1). β-actin served as loading control and is depicted for each individual Western blot membrane.

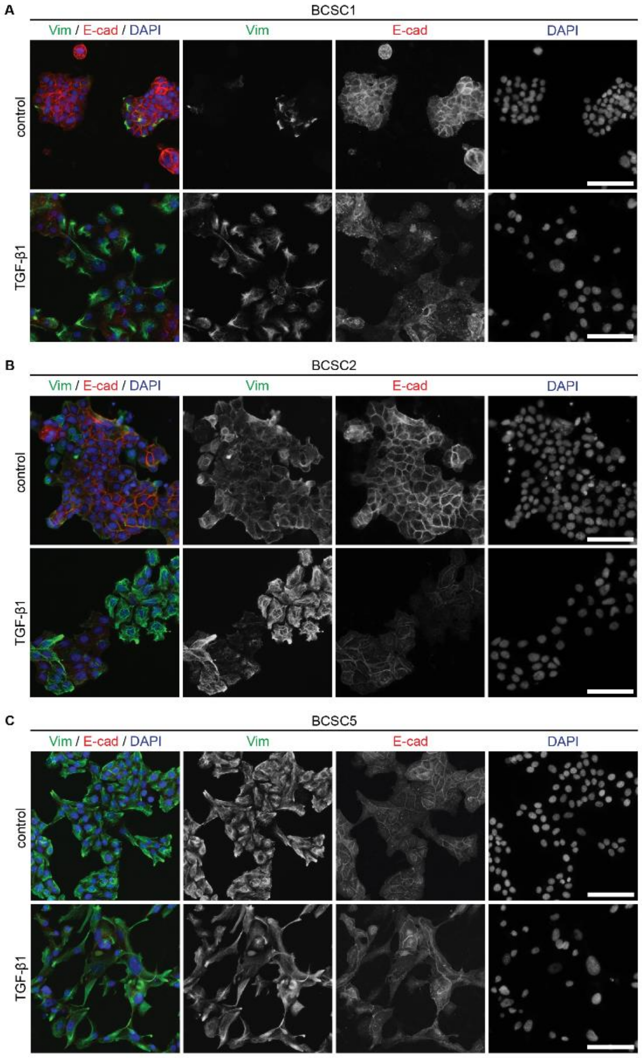

Figure S9. E-cadherin and vimentin expression of BCSCs after TGF-β1 stimulation. Immunofluorescence analysis of BCSC1 (**A**), BCSC2 (**B**), and BCSC5 (**C**) using antibodies against epithelial E-cadherin (red) and mesenchymal vimentin (green). Cell nuclei were counterstained with DAPI. Scale bars represent 100 μm.

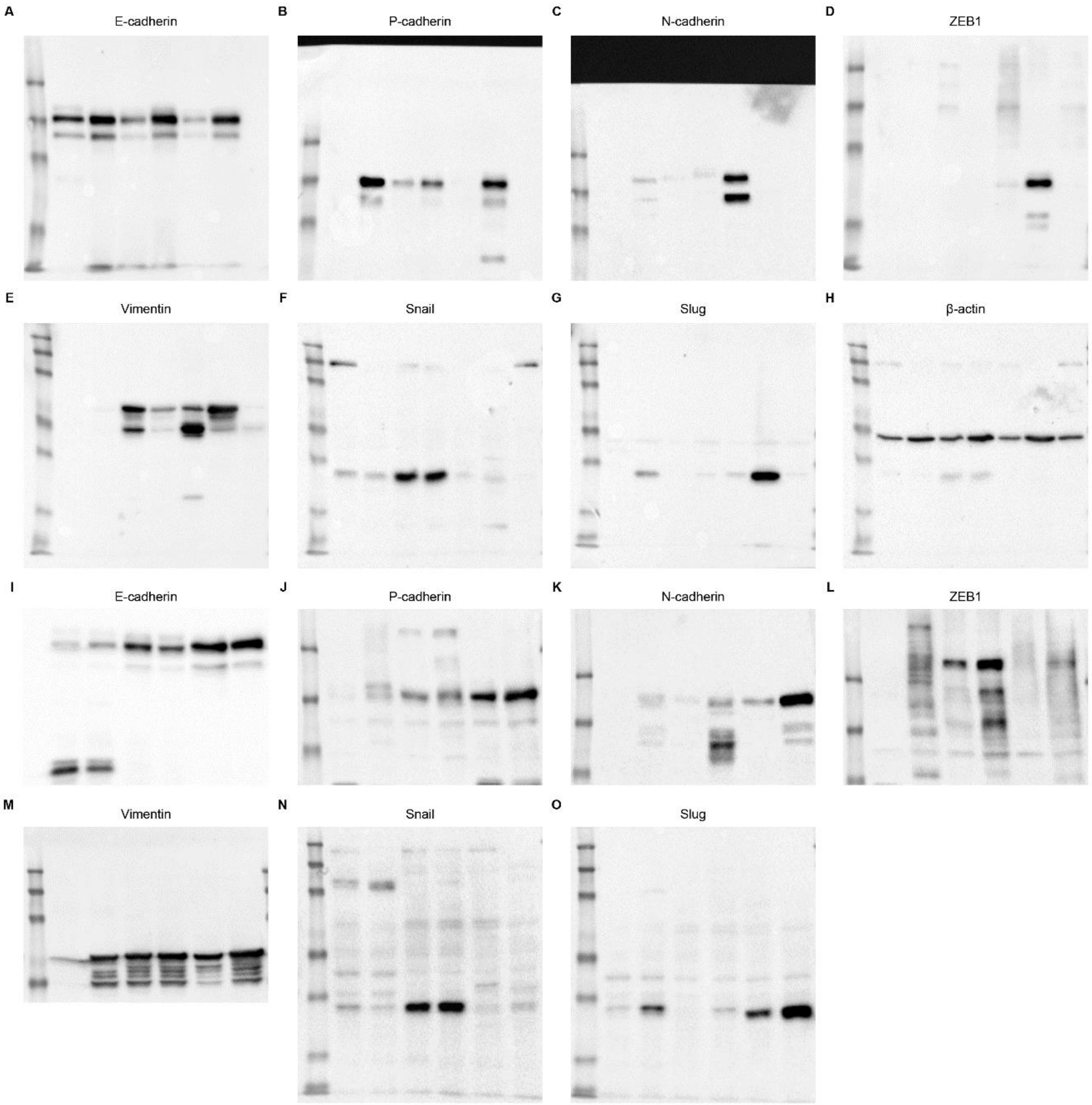

Figure S10. Uncropped Western blots. Depicted are the uncropped Western blots for Figure 3K (**A**-**H**) and Supplementary Figure S8 (**I**-**O**).

A

	MCF7	BCSC1	BCSC2	BCSC3	BCSC4	BCSC5	MDA-MB-231
E-cadherin	3.58	4.11	0.74	1.02	0.78	0.89	ND
N-cadherin	ND	0.26	0.08	0.05	8.34	ND	ND
ZEB1	ND	ND	0.57	ND	7.23	ND	0.36
P-cadherin	ND	1.05	0.36	0.80	ND	0.80	ND
Snail	0.39	0.17	1.78	0.87	0.16	0.14	ND
Slug	ND	0.24	ND	0.02	0.11	1.45	ND
Vimentin	ND	ND	0.91	0.19	0.33	0.98	ND

B

	BCSC1		BCSC2		BCSC5	
TGF-β1	-	+	-	+	-	+
E-cadherin	0.19	0.25	1.06	0.99	1.79	1.33
N-cadherin	0.01	0.15	0.05	0.56	0.30	1.70
P-cadherin	0.04	0.26	0.45	0.63	0.75	1.23
ZEB1	0.01	0.76	0.70	1.49	0.22	0.72
Vimentin	0.04	0.65	1.79	1.43	0.84	0.92
Snail	0.26	0.14	2.50	1.81	0.15	0.16
Slug	0.10	0.58	0.08	0.14	0.72	1.10

Figure S11. Densitometry readings/intensity ratios relative to β-actin. Depicted are the densitometry readings/intensity ratios relative to the corresponding β-actin expression level for the Western blots shown in Figure 3K (**A**) and Supplementary Figure 8 (**B**). The data was analyzed using ImageJ.

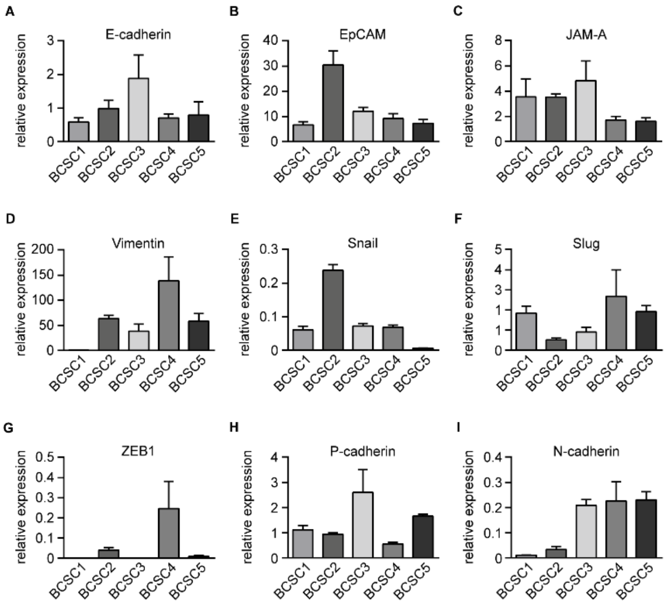

Figure S12. BCSCs co-express epithelial and mesenchymal markers in 3D cultures. (**A**–**I**) Analysis of EMT-related gene expression in BCSCs cultured in 50% Matrigel at the mRNA level using qRT-PCR. Shown are mRNA levels of E-cadherin (**A**), EpCAM (**B**), JAM-A (**C**), Vimentin (**D**), Snail (**E**), Slug (**F**), ZEB1 (**G**), P-cadherin (**H**), and N-cadherin (**I**) relative to HPRT1 (n≥3). Data represents means + SEM.

Table S1. List of antibodies.

	Antigen	Application	Manufacturer	Dilution
Primary Antibodies	CD24	Flow cytometry	eBioscience (46-0247)	1:100
	CD44	Flow cytometry	eBioscience (12-0441-81)	1:000
	CD49f	Flow cytometry	eBioscience (46-0495)	1:200
	E-cadherin	IHC	Dako (IR059)	RTU[1]
	E-cadherin	IF	BD Biosciences (610182)	1:400
		WB		1:5000
	EpCAM	Flow cytometry	eBioscience (660 50-9326)	1:100
	ER	IHC	Dako (IR084)	RTU[1]
	HER2	IHC	Dako (A0485)	1:350
	Keratin 14	IF	Covance (PRB-155P)	1:250
	Keratin 18	IF	Dako (M7010)	1:250
	Keratin 5	IF	Covance (PRB-160P)	1:250
	Keratin 5/6	IHC	Dako (IR780)	RTU
	Keratin 8	IF	Sigma-Aldrich (C5301)	1:250
	Keratin 8/18	IHC	Dako (IR094)	RTU[1]
	Ki67	IHC	Dako (IR626)	RTU[1]
	N-cadherin	WB	Cell Signaling Technology (13116)	1:1000
	P-cadherin	WB	Cell Signaling Technology (2130)	1:1000
	PR	IHC	Dako (IR068)	RTU[1]
	Slug	WB	Cell Signaling Technology (9585)	1:1000
	Snail	WB	Cell Signaling Technology (3879)	1:1000
	Vimentin	IHC	Dako (IR630)	RTU[1]
	Vimentin	IF	GeneTex (GTX16700)	1:50
		WB		1:1000
	ZEB1	WB	Sigma-Aldrich (HPA027524)	1:5000
	β-actin	WB	Sigma-Aldrich (A5441)	1:5000
Secondary Antibodies	Donkey anti-mouse IgG (H+L) Alexa Fluor 568	IF	Life Technologies (A10037)	1:250
	Donkey anti-rabbit IgG (H+L) Alexa Fluor 488	IF	Life Technologies (A21206)	1:250
	Goat anti-mouse IgG (H+L) HRP	WB	Thermo Fisher Scientific (31430)	1:25000
	Peroxidase-AffiniPure Goat anti-rabbit IgG (H+L)	WB	Dianova (111-035-144)	1:25000

[1] RTU = ready-to-use.

Table S2. Oligonucleotides for qRT-PCR. All oligonucleotides are listed from 5′→3′ and were purchased from Sigma-Aldrich or Eurofins.

Gene	Forward (5′-3′)	Reverse (5′-3′)	UPL
E-cadherin	cccgggacaacgtttattac	gctggctcaagtcaaagtcc	35
EpCAM	ccatgtgctggtgtgtgaa	tgtgttttagttcaatgatgatcca	3
HPRT1	tgaccttgatttattttgcatacc	cgagcaagacgttcagtcct	73
JAM-A	tcaaggtcaagctcatcgtg	ggcagaggaggggatgtta	58
N-cadherin	agtatccggtccgatctgc	ctgtggggtcattgtcagc	80
P-cadherin	gctggggaaagtattcatgg	cctttccttcagtgaccttcttt	61
Slug	tggttgcttcaaggacacat	gcaaatgctctgttgcagtg	7
Snail	gctgcaggactctaatccaga	atctccggaggtgggatg	11
TGF-β1	actactacgccaaggaggtcac	tgcttgaacttgtcatagatttcg	31
TGF-β2	acaacaccctctggctcagt	tagaaagtgggcgggatg	50
TGF-β3	aagaagcgggctttggac	cgcacacagcagttctcc	38
TGF-βR1	aaattgctcgacgatgttcc	cataataaggcagttggtaatcttca	31
TGF-βR2	caccgcacgttcagaagtc	tggatgggcagtcctattaca	43
TGF-βR3	gatttcatcttcggcttgaaa	gctcaggaggaatagtgtgga	82
Vimentin	gaccagctaaccaacgacaaa	gaagcatctcctcctgcaat	39
ZEB1	aactgctgggaggatgacac	tcctgcttcatctgcctga	57
